# On-treatment serum albumin levels can predict 28-day mortality and guide albumin infusion in sepsis patients

**DOI:** 10.3389/fmed.2025.1490838

**Published:** 2025-05-14

**Authors:** Danfeng Ren, Xiangyun Dang, Tianzhi Ni, Jingwen Zhou, Ze Zhang, Shan Fu, Wentao Zhang, Taotao Yan, Yingren Zhao, Jinfeng Liu

**Affiliations:** ^1^Department of Infectious Diseases, The First Affiliated Hospital of Xi’an Jiaotong University, Xi’an, China; ^2^Shaanxi Clinical Medical Research Center for Infectious Diseases, Xi’an, China

**Keywords:** sepsis, serum albumin, target level, mortality, sepsis shock

## Abstract

**Background:**

As the most abundant protein in plasma, albumin (ALB) presents close association with prognosis of septic patients. Whereas, the benefit and the target level of ALB infusion remain controversial.

**Methods:**

We conducted a retrospective investigation to assess whether on-treatment ALB levels could predict 28-day mortality and try to identify the optimal level for ALB infusion. All patients diagnosed as sepsis from January 2016 to December 2020 were recruited and re-evaluated using Sepsis-3 criteria.

**Results:**

A total of 199 eligible patients were enrolled in this study. Compared with the survival group, the non-survival group had more males (73.97 vs. 56.35%), older patients (62.78 ± 15.93 vs. 56.43 ± 18.46), and a higher proportion of Gram-positive bacterial infection (27.40 vs. 23.02%), higher Sequential Organ Failure Assessment (SOFA) score (7.00–13.00 vs. 6.00–12.00), higher APACHE II score (18.25–29.00 vs. 15.00–26.00), higher PCT (2.84–49.18 vs. 2.43–19.14), more patients with septic shock (65.75%vs. 43.65%), shorter ICU-stay days (11.04 ± 6.28 vs. 14.83 ± 8.58), longer mechanical ventilation days (7.23 ± 7.07 vs. 5.04 ± 8.52), with statistically significant differences (*p* < 0.050). Furthermore, we identified that the ALB level on day 7 (HR, 0.920; 95% CI, 0.847 to 0.999; *p* = 0.046) and the maximum ALB level within the first 14 days (HR, 0.900; 95% CI, 0.838 to 0.967; *p* = 0.004) were independent protective factor for the 28-day prognosis in septic patients. Moreover, ROC curve analysis indicated that optimal target level for first 14-day maximum and on day 7 were 33.45 g/L and 27.85 g/L, respectively. Correspondingly, a negative correlation between ALB level and mortality was defined with Kaplan–Meier survival curve analysis. Further subgroup analysis showed that the group with ALB above the cut-off value was associated with favorable outcomes in female patients under 60 years, with SOFA score less than 7, and APACHE II score less than 19.

**Conclusion:**

ALB levels on day 7 and the maximum ALB level within first 14 days after ICU admission were closely associated with 28-day mortality. 27.85 g/L would work as the target level of ALB infusion on 7 day to improve the prognosis of sepsis patients.

## Introduction

Sepsis is a life-threatening clinical condition defined as organ dysfunction caused by a dysregulated host response to infection (1). It has long been identified as one of the leading causes of mortality, especially among hospitalized patients in Intensive Care Unit (ICU) (2). Based on data from the World Health Organization (WHO), sepsis is responsible for approximately 6 million global fatalities annually and imposes a substantial economic burden (3). Despite extensive use of the “Surviving Sepsis Campaign Guidelines”, mortality in septic patients is approximately 30%, notably who suffered septic shock remain unacceptably high (4, 5). In the USA, for example, sepsis is a leading cause of in-hospital deaths and accounts for more than USD 20 billion in annual hospital expenditures (6). Despite treatment according to the current management recommendations, it is of great importance to improve therapeutic efficacy and develop innovative therapeutic strategies for this thorny disease.

While the pathogenesis of sepsis is multifactorial and incompletely understood, the uncontrolled production of inflammatory mediators is strongly associated with organ dysfunction and poor prognosis (7–9). The Surviving Sepsis Campaign (SSC) has established and endorsed international clinical practice guidelines for the management of sepsis (10, 11). Fluid therapy is the cornerstone in the hemodynamic resuscitation of patients with sepsis and septic shock (10, 12). Timely and effective restoration of plasma volume is crucial for the reversal of tissue hypoxia and the maintenance of organ homeostasis. Crystalloids have been identified as the major initial resuscitation fluid, whereas, there are controversies regarding infusions of plasma and Albumin (ALB).

ALB, a 66.5-kDa protein synthesized in the liver (13), accounting for 60% of the total plasma protein, plays key roles in protecting vascular endothelial integrity by protecting endothelial glycocalyx, maintaining 70 to 80% of effective plasma colloid osmotic pressure. It also possesses various physiological functions, such as antioxidant and anti-inflammatory activities, immunomodulatory, maintaining the acid–base balance, and participating in the transport, distribution, and metabolism of a variety of endogenous and exogenous substances (14–16). These diverse features of ALB render it an essential role in critically ill patients, especially those with sepsis. In addition, serum ALB level has been identified as an independent risk factor for increased short- and long-term mortality in patients with acute conditions such as trauma, cardiogenic shock, and sepsis (17). Hypoalbuminemia in sepsis arises from multifactorial mechanisms, including capillary leakage due to endothelial injury, reduced hepatic synthesis from inflammatory cytokine suppression, and increased catabolism and loss.

The use of ALB in sepsis presents contrasting effects and remains controversial. It is noteworthy that ALB has been administered for many decades for fluid resuscitation and is considered safe in critically ill patients (18). An earlier investigation reported decreasing mortality in patients receiving ALB infusion compared to those receiving crystalloids (19). Whereas, no consensus has been reached on the benefit of ALB over crystalloids when concerning mortality. The SAFE study showed a 28-day mortality reduction owing to ALB infusion, outperforming normal saline (20). However, this benefit was not confirmed in the ALBIOS study comparing the use of 20% ALB and crystalloid solution versus crystalloid solution alone (18, 21). International guidelines for the management of sepsis and Septic Shock 2021 state that a combination of ALB rather than crystalloids fluid alone is recommended in adult patients requiring resuscitation with large doses of crystalloids fluid (10). In hypoalbuminemia patients, ALB infusion would be expected to improve outcomes. Nevertheless, there is still no universal recommendation regarding the concentration to select, dosage, timing of administration, and target level.

In 2016, a new definition of sepsis (Sepsis-3) was developed, and the population of septic patients correspondingly changed. To investigate the association of ALB level with outcomes in patients meeting the criteria for Sepsis-3, and further evaluate the optimal target level of ALB infusion, we conducted this retrospective cohort study and the results would provide suggestions for clinical practice.

## Patients and methods

### Study population

This retrospective study was conducted in the First Affiliated Hospital of Xi’an Jiaotong University (a large tertiary referral hospital located in Xi’an, Shaanxi, Northwest China) with the approval of the institutional ethics review committee. Patients diagnosed with sepsis were screened by two independent reviewers from January 2016 to December 2020. The diagnosis of sepsis was defined according to the Third International Consensus Definition for Sepsis and Septic Shock (Sepsis-3) (1, 22). Septic patients older than 18 years and hospital stay time of more than 72 h were enrolled. The main exclusion criteria included patients with chronic liver and renal insufficiency, malnutrition, pregnant women, or nursing women. All patients received standard medical treatment as per the guidelines outlined by the Surviving Sepsis Campaign, which encompasses antimicrobial therapy, fluid therapy, glucose control, supportive care, and nutrition supplements (22, 23).

The Ethics Committee of The First Affiliated Hospital of Xi’an Jiaotong University waived the need for informed consent because this study used a retrospective and anonymous dataset and approved this study (No. XJTU1AF2021LSK-286).

### Data collection

Demographic, clinical, and laboratory data were retrieved from the electronic medical records. Demographic data included age and sex. Clinical data included comorbidities, complications, pathogens, sites of infection, sequential organ failure assessment (SOFA) score, acute physiology and chronic health evaluation (APACHE II) score, and inpatient days.

Laboratory parameters included routine blood analyses, kidney function, liver function, coagulation function, C-reactive protein (CRP), procalcitonin (PCT), lactic acid (Lac), and erythrocyte sedimentation rate (ESR), etc.; these tests were carried out routinely by auto-analyzers. The neutrophil-to-lymphocyte ratio (NLR) and systemic immune-inflammatory index (SII) were calculated based on routine blood analysis. The NLR is a biomarker derived from a routine complete blood count, which is a simple, cost-effective tool to assess systemic inflammation and immune status and can aid in risk stratification and prognosis in sepsis. The SII was calculated as Neutrophil count × Platelet count / Lymphocyte count, which reflects systemic inflammation and immune dysregulation, with higher values indicating exacerbated inflammatory responses. Laboratory parameters were dynamically recorded, including day 0, day 1, day 3, day 7, day 14, and day 28. We defined the day with a suspected infection combined with an available acute increase in SOFA score ≥ 2 from baseline as “Day 0.” Management and clinical outcomes were also recorded.

### Outcome measures

The primary outcome was in-hospital mortality. The secondary outcome was 28-day mortality.

### Statistical analysis

Statistical analysis was carried out using SPSS 26.0 for Windows (SPSS Inc., Chicago, IL, United States). Continuous variables were expressed as means (± standard deviation) or median (range). All variables were checked for normality of distribution by Kolmogorov–Smirnov’s test. Continuous variables were compared using the *t-test* or Mann–Whitney *U* test. Categorical variables were expressed as frequencies and percentages, and the Chi-squared test or Fisher’s exact test was applied to compare the two groups of patients. Logistic regression analysis was applied to assess the clinical correlations. ROC curve, sensitivity, and specificity were used to find the best threshold of indicators, and Kaplan–Meier survival curve analysis was used to calculate the value of each indicator in predicting 28-day mortality. All statistical tests were two-tailed, *p* values < 0.05 were considered statistically significant, and all confidence intervals were at the 95% level. Charts were drawn using GraphPad Prism 8.0.

## Results

### Baseline characteristics of participants

**Figure 1:**
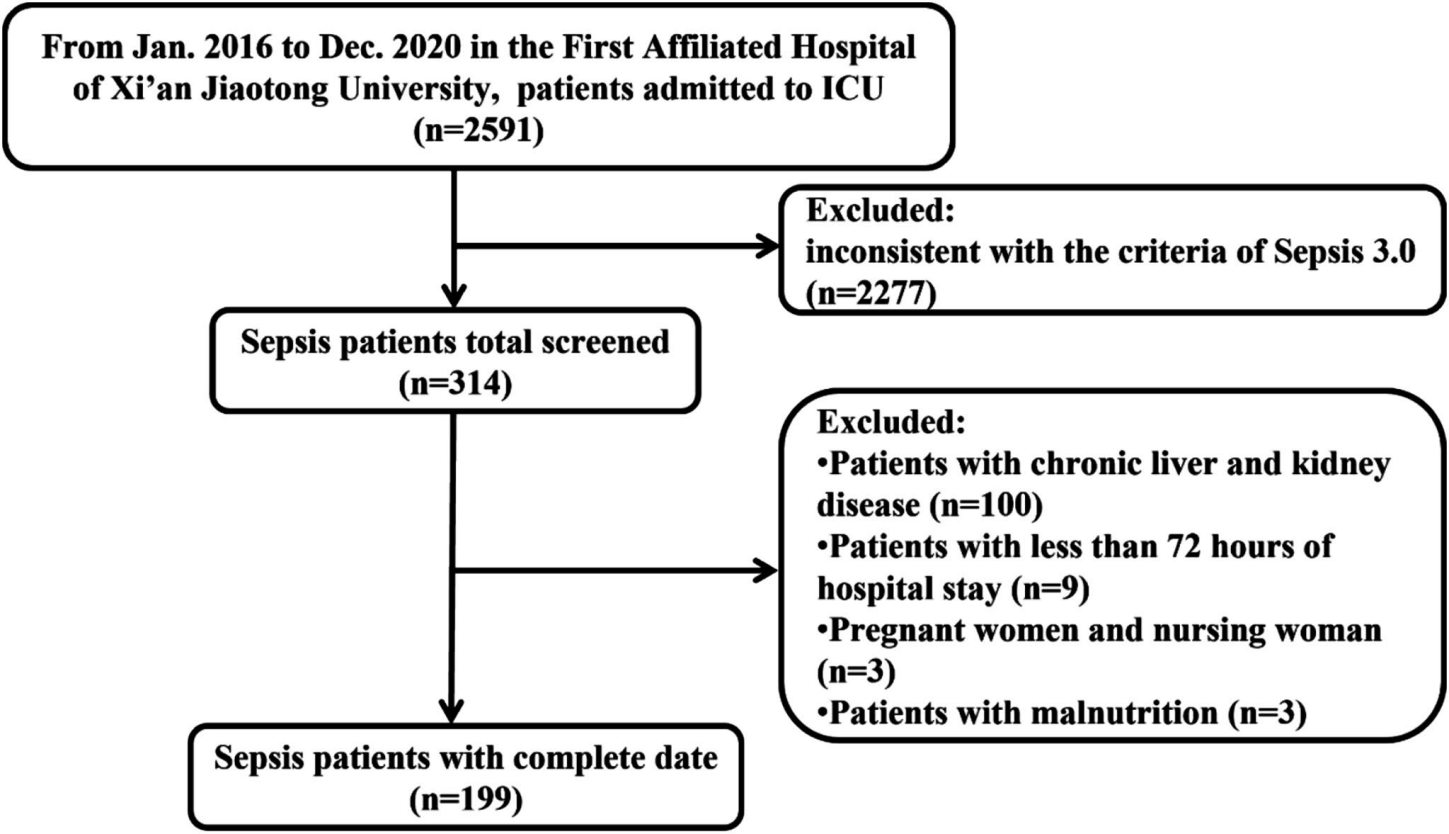
Flowchart of included patients.

As shown in the flowchart of patient selection, a total of 2,591 septic patients were reviewed from January 2016 to December 2020 (Figure 1). According to the inclusion and exclusion criteria, we enrolled 199 eligible patients for final analysis. Among them, 96 patients experienced only sepsis, and 103 fulfilled the septic shock criteria after re-evaluating these patients according to the Sepsis-3 criteria. Overall, 73 cases died (36.68%), and the mortality rate was 46.60% in the septic shock cohort.

The baseline characteristics of the patients at the time of admission to the ICU and major clinical outcomes were presented in Table 1. The frequency of comorbidities, concurrent infection, and infection sites were comparable between the survival and non-survival groups. The non-survival group had more males (73.97 vs. 56.35%, *p* = 0.013) and older patients (62.78 ± 15.93 vs. 56.43 ± 18.46, *p* = 0.015) and was presented with a higher proportion of Gram-positive bacterial infection (27.40 vs. 23.02%, *p* = 0.000). It should be noted that SOFA score (7.00–13.00 vs. 6.00–12.00, *p* = 0.004), APACHE II score (18.25–29.00 vs. 15.00–26.00, *p* = 0.005), PCT (2.84–49.18 vs. 2.43–19.14, *p* = 0.006), and incidence of septic shock (65.75 vs. 43.65%, *p* = 0.003) were significantly higher in the non-survival group than in the survival group. Compared to the survival group, the non-survival group had shorter ICU-stay days (11.04 ± 6.28 vs. 14.83 ± 8.58, *p* = 0.000) and longer mechanical ventilation days (7.23 ± 7.07 vs. 5.04 ± 8.52, *p* = 0.000). All patients were treated with ALB in the study.

**Table 1:** Baseline characteristics and clinical outcomes of enrolled patients.

Variables	Total (*n* = 199)	Survival group (*n* = 126)	Non-survival group (*n* = 73)	*p*-value
Demographics				
Gender, male (*n*, %)	125(62.81)	71(56.35)	54(73.97)	0.013
Age, years	58.76 ± 17.80	56.43 ± 18.46	62.78 ± 15.93	0.015
Comorbidities, *n* (%)				
Hypertension	54(27.14)	31(24.60)	23(31.51)	0.291
Diabetes	35(17.59)	21(16.67)	14(19.18)	0.654
Concurrent infection, *n* (%)	112(94.12)	73(57.94)	39(53.42)	0.536
Infection sites, *n* (%)				
Pneumonia	155(77.89)	94(74.60)	61(83.56)	0.142
Gastrointestinal	82(41.21)	58(46.03)	24(32.88)	0.069
Skin and soft tissue infection	24(12.06)	13(10.32)	11(15.07)	0.321
Bloodstream infection	37(18.59)	21(16.67)	16(21.92)	0.359
Urinary tract infection	37(18.59)	26(20.63)	11(15.07)	0.331
Multi-site infection(≥2)	110(55.28)	72(57.14)	38(52.05)	0.565
Infection pathogen, *n* (%)				
G + bacteria	49(24.62)	29(23.02)	20(27.40)	0.000
G- bacteria	96(48.24)	65(51.59)	31(42.47)	0.215
Fungus	46(23.12)	33(26.19)	13(17.81)	0.176
Virus	13(6.53)	8(6.35)	5(6.85)	1.000
Multi-pathogen infection (≥2)	89(44.72)	52(41.27)	37(50.68)	0.198
Severity scores				
SOFA score	6.00–12.00	6.00–12.00	7.00–13.00	0.004
APACHE II score	16.00–27.00	15.00–26.00	18.25–29.00	0.005
Laboratory tests				
PCT, ng/ml	2.73–40.64	2.43–19.14	2.84–49.18	0.006
Lac, mmol/L	1.20–3.58	1.00–3.14	1.40–5.00	0.075
CRP, mg/L	78.38–215.60	83.23–224.78	69.35–204.70	0.232
HGB, g/L	84.75–119.00	90.50–127.00	79.00–117.00	0.012
WBC, ×10^9^/L	7.67–19.76	7.90–20.72	7.09–18.50	0.813
NEUT, ×10^9^/L	6.37–17.40	6.83–18.07	5.60–17.17	0.840
LYM, ×10^9^/L	0.35–0.94	0.35–0.94	0.34–1.02	0.728
PLT, ×10^9^/L	45.00–159.00	48.25–156.75	40.00–165.75	0.777
NLR	9.07–32.03	9.62–32.58	8.10–28.34	0.291
SII	657.12–3601.70	677.69–3985.83	565.06–3318.29	0.899
INR	1.21–1.64	1.18–1.63	1.27–1.68	0.077
D-dimer, mg/L	2.99–13.36	2.75–11.97	3.50–19.74	0.161
ALT, IU/L	21.00–98.00	22.00–85.00	19.25–89.50	0.391
TBIL, umol/L	14.15–44.80	14.10–43.25	13.60–54.58	0.573
ALB, g/L	24.40–31.00	24.80–31.05	23.45–30.65	0.668
Complications				
Septic shock, *n* (%)	103(51.76)	55(43.65)	48(65.75)	0.003
AKI, *n* (%)	64(32.16)	35(27.78)	29(39.73)	0.082
Clinical outcomes				
ICU-stay days	13.44 ± 8.01	14.83 ± 8.58	11.04 ± 6.28	0.000
Mechanical ventilation days	5.84 ± 8.07	5.04 ± 8.52	7.23 ± 7.07	0.000

Values reported with mean ± standard deviation (SD), number (percentage), or median (interquartile range). G+, Gram-positive; G-, Gram-negative; SOFA, sequential organ failure assessment; APACHE, acute physiology and chronic health evaluation; PCT, procalcitonin; Lac, lactic acid; CRP, C-reactive protein; HGB, hemoglobin; WBC, white blood cell; NEUT, neutrophile granulocyte; LYM, lymphocyte; PLT, platelet; NLR, neutrophil-to-lymphocyte ratio; SII, systemic immune-inflammatory index; INR, international normalized ratio; ALT, alanine transaminase; TBIL, total bilirubin; ALB, albumin; AKI, acute kidney injury; ICU, intensive care unit. Reference values, PCT (<0.05) ng/ml; CRP (0–10)m/L; HGB (110–150) g/L (female), (130–175) g/L (male); WBC count (4.0–10) × 10^9^/L; NEUT (1.8–6.3) × 10^9^/L; LYM count (1.1–3.2) × 10^9^/L; PLT (125–350) × 10^9^/L; INR (0.94–1.24); D-dimer (0-1)mg/L; ALT (9–40) U/L; TBIL (3.4–17.1) μmol/L; ALB (40-55)g/L.

### Risk factors associated with 28-day mortality in patients with sepsis

To further analyze the risk factors associated with the 28-day mortality in septic patients, we performed univariable Cox regression analysis using a range of variables, including gender, age, comorbidities, concurrent infection, APACHE II score, SOFA score, PCT, interleukin-6 (IL-6), lac, CRP, white blood cell (WBC) counts, neutrophile granulocyte (NEUT) counts, platelet (PLT) counts, hemoglobin (HGB), NLR, SII, international normalized ratio (INR), D-dimer, alanine transaminase (ALT), total bilirubin (TBIL), ALB level, acute kidney injury (AKI) and septic shock (Table 2). Cox regression analysis demonstrated that gender (HR, 0.454, 95% CI, 0.242–0.853, *p* = 0.014), age (HR, 1.021, 95% CI, 1.004–1.039, *p* = 0.016), baseline APACHE II scores (HR, 1.053, 95% CI, 1.015–1.093, *p* = 0.006), baseline SOFA scores (HR, 1.105, 95% CI, 1.030–1.185, *p* = 0.005), PCT (HR, 0.998, 95% CI, 0.979–0.998, *p* = 0.017), PLT counts (HR, 0.000, 95% CI, 0.000–0.176, *p* = 0.019), and incidence of septic shock (HR, 2.479, 95% CI,1.363–4.507, *p* = 0.003) were identified as significant risk factors for 28-day mortality in patients with sepsis. Male, older, and higher baseline PCT, APACHE II, and SOFA scores were associated with 28-day adverse outcomes. Since baseline ALB levels were associated with disease prognosis as observed in previous studies, we next investigated the association of ALB levels with prognosis for 28-day survival. To further analyze the association of ALB levels with disease prognosis, we performed univariable Cox regression analysis using ALB levels at each time point and period. As shown in Table 2, ALB levels on day 7 (HR, 0.920, 95% CI, 0.847–0.999, *p* = 0.046) and day 14 (HR, 0.893, 95% CI, 0.802–0.995, *p* = 0.040) and the maximum ALB level within the first 14 days (HR, 0.900, 95% CI, 0.838–0.967, *p* = 0.004) were closely associated with 28-day mortality, working as a protective factor for 28-day prognosis.

**Table 2:** Univariate analyses of the Cox proportional hazards model for risk of 28-day mortality.

Variables	Univariable Cox regression	
	HR (95% CI)	*p*-value
Gender	0.454 (0.242,0.853)	0.014
Age, years	1.021 (1.004,1.039)	0.016
Diabetes Mellitus	1.456 (0.689,3.079)	0.325
Hypertension	1.742 (0.921,3.294)	0.088
Concurrent infection	1.008 (0.978,1.039)	0.601
APACHE II score	1.053 (1.015,1.093)	0.006
SOFA score	1.105 (1.030,1.185)	0.005
PCT	0.998 (0.979,0.998)	0.017
IL-6	1.000 (1.000,1.001)	0.360
Lactic acid	1.095 (0.994,1.207)	0.066
CRP	1.000 (1.000,1.000)	0.556
WBC	0.997 (0.993,1.001)	0.191
NEUT	0.996 (0.966,1.028)	0.808
PLT	0.000 (0.000,0.176)	0.019
HGB	1.008 (0.996,1.019)	0.188
NLR	1.003 (0.998,1.008)	0.297
SII	1.000 (1.000,1.000)	0.418
INR	1.012 (0.999,1.025)	0.066
D-dimer	0.899 (0.775,1.043)	0.162
ALT at admission	0.998 (0.994,1.002)	0.397
TBIL at admission	1.000 (1.000,1.000)	0.578
ALB at admission	1.032 (0.958,1.112)	0.405
ALB (day 7)	0.920 (0.847,0.999)	0.046
ALB (day 14)	0.893 (0.802,0.995)	0.040
Max ALB (day 0–7)	0.933 (0.870,1.000)	0.051
Max ALB (day 0–14)	0.900 (0.838,0.967)	0.004
Min ALB (day 0–7)	0.991 (0.914,1.075)	0.829
Min ALB (day 0–14)	0.986 (0.907,1.071)	0.739
AKI	0.954 (0.514,1.770)	0.881
Septic shock	2.479 (1.363,4.507)	0.003

APACHE, acute physiology and chronic health evaluation; SOFA, sequential organ failure assessment; PCT, procalcitonin; IL-6, interleukin-6; CRP, C-reactive protein; WBC, white blood cell; NEUT, neutrophile granulocyte; PLT, platelet; HGB, hemoglobin; NLR, neutrophil-to-lymphocyte ratio; SII, systemic immune-inflammatory index; INR, international normalized ratio; ALT, alanine transaminase; TBIL, total bilirubin; ALB, albumin; Max, maximum; Min, minimum; ALB (day 7), serum albumin on day 7 after admission to ICU; ALB (day 14), serum albumin on day 14 after admission to ICU; Max ALB (day 0–7), the highest serum albumin within 7 days after admission to ICU; Max ALB (day 0–14), the highest serum albumin within 14 days after admission to ICU; Min ALB (day 0–7), minimum serum albumin within 7 days after admission to ICU; Min ALB (day 0–14), minimum serum albumin within 14 days after admission to ICU; AKI, acute kidney injury. Reference values, PCT (<0.05) ng/ml; IL-6 (0–7) pg./mL; CRP (0–10)m/L; WBC count (4.0–10) × 109/L; NEUT (1.8–6.3) × 109/L; PLT (125–350) × 109/L; HGB (110–150) g/L (female), (130–175) g/L (male); INR (0.94–1.24); D-dimer (0-1)mg/L; ALT (9–40) U/L; TBIL (3.4–17.1) μmol/L; ALB (40–55) g/L.

### Association between ALB levels and 28-day survival

**Figure 2:**
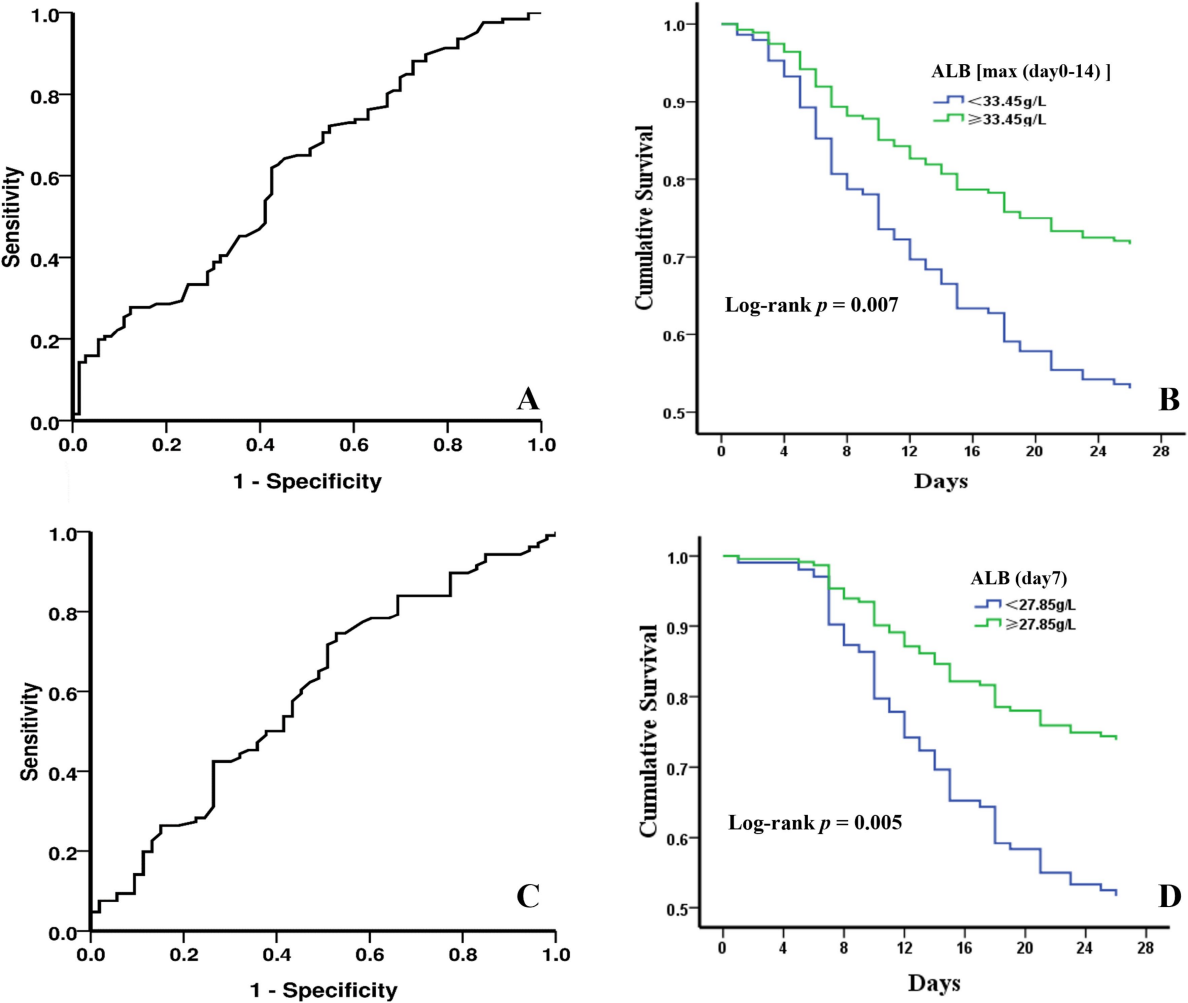
The predictive value of ALB levels by ROC analysis and 28-day cumulative survival probabilities under different ALB levels with Kaplan–Meier analysis. **(A)** 28-day mortality diagnostic value of 14-day maximum ALB level. **(B)** survival curves of sepsis patients divided by the optimal cut-off value for 14-day maximum ALB level. **(C)** 28-day mortality diagnostic value of ALB level on day 7. **(D)** survival curves of sepsis patients divided by the optimal cut-off value for ALB level on day 7.

The ROC curve analysis of ALB levels and poor outcomes at 28-day follow-up was shown in Figures 2A,C. The overall bootstrapped time-dependent C-statistics were 14-day maximum ALB level at 0.609 (95% CI, 0.527–0.690, *p* = 0.011) and ALB level on day 7 at 0.598 (95% CI, 0.503–0.693, *p* = 0.044). ROC curve analysis indicated that optimal cut-off values for the 14-day maximum ALB level and ALB level on day 7 were 33.45 g/L and 27.85 g/L, respectively. The predictive sensitivity and specificity were 61.90 and 57.50% for the 14-day maximum ALB level, and for the ALB level on day 7, they were 74.50 and 47.20%. Kaplan–Meier survival curves estimated by Cox proportional hazard regression in the groups with different ALB levels were presented in Figures 2B,D. The patients with maximum ALB level ≥ 33.45 g/L within 14-day exhibited a lower 28-day mortality rate than those with ALB levels lower than 33.45 g/L (HR, 1.901, 95%CI, 1.194–3.025; log-rank *p* = 0.007]. Similarly, the patients with ALB level ≥ 27.85 g/L on day 7 had a lower 28-day mortality rate than those with ALB level < 27.85 g/L (HR, 2.172, 95%CI, 1.266–3.727; log-rank *p* = 0.005]. More significantly, the earlier the ALB level is raised to a certain level, the higher the 28-day survival rate of patients with sepsis.

### Changes in inflammatory indexes under different ALB levels

The pathological mechanism of sepsis is complex, mainly due to the inflammation disorder; excessive inflammation will cause obstacles to organ function. In addition, ALB has shown anti-inflammatory effects. To investigate the inflammatory response under different ALB levels, we analyzed the dynamic changes of symptom inflammatory indicators under different ALB levels.

**Figure 3:**
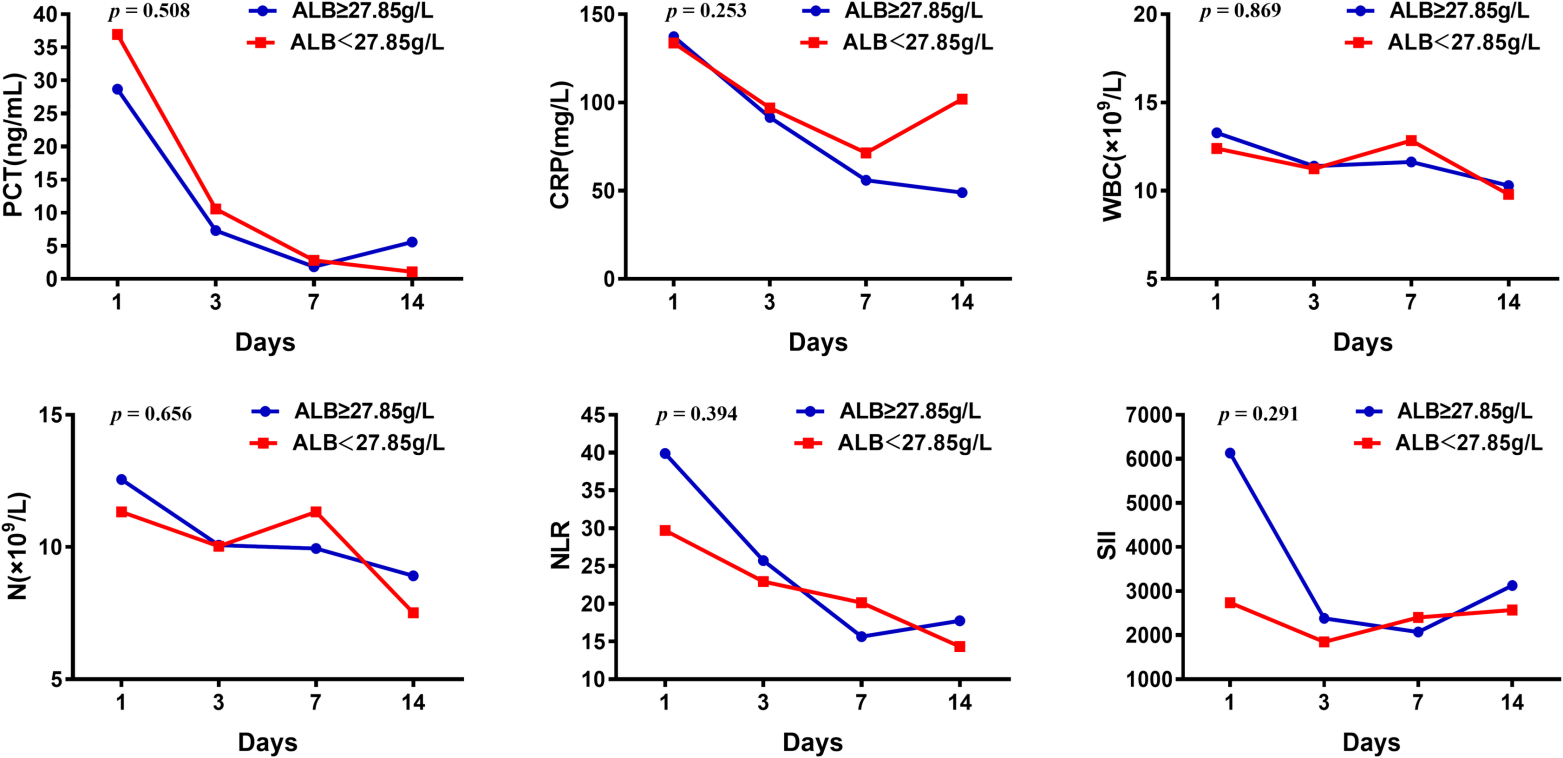
Changes of inflammatory indicators in septic patients with ALB higher or less than 27.85 g/L.

Univariate Cox regression analysis showed that ALB level on day 7 after ICU admission was associated with the 28-day prognosis of sepsis patients, and the optimal cut-off value was 27.85 g/L. Patients were categorized into two groups according to the optimal cut-off value, and the changes in inflammatory indicators were compared (Figure 3). The level of PCT, CRP, WBC counts, NEUT, NLR, and SII in both groups showed downward trends in the first 14 days, indicating reduced inflammatory response. However, there was no statistical difference between the two groups. When septic patients were grouped according to the optimal cut-off value of the maximum ALB level within 14 days, the above indicators showed similar trends (Supplementary Figure S1). Dynamic changes in CRP and NLR values displayed significant differences between the two groups, and the group with ALB ≥33.45 g/L appeared to decrease faster.

### Comparison of short-term prognosis in patients with different ALB levels

**Figure 4:**
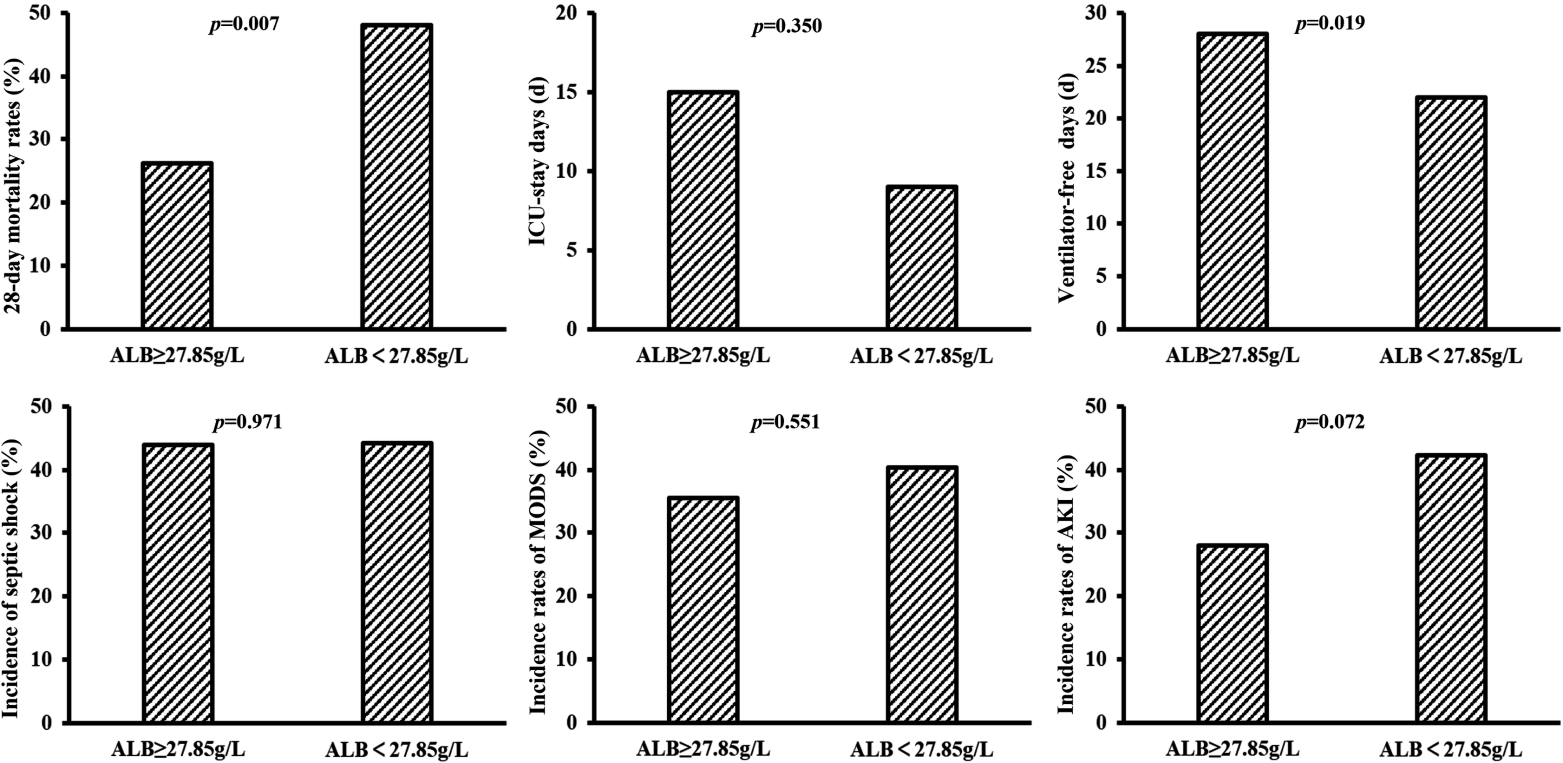
The predictive value of ALB levels on day 7 for clinical prognosis.

The 28-day mortality rate in the group with ALB level ≥ 27.85 g/L on day 7 was significantly lower (*p* = 0.007) and with a longer duration of ventilator-free days (*p* = 0.019). Additionally, a lower incidence of septic shock, multiple organ dysfunction syndrome (MODS), and AKI and a longer duration of ICU-stay days had been observed in patients with ALB level ≥27.85 g/L on day 7, although these differences were not statistically significant between the two groups (Figure 4). Similarly, the group with a 14-day maximum ALB level ≥33.45 g/L had a lower 28-day mortality rate and longer ICU-stay days (*p* = 0.008 & *p* = 0.020), whereas, no obvious differences were observed in the incidence of septic shock, ventilator-free days, and incidence of MODS and AKI between the two groups with 14-day maximum ALB levels ≥33.45 g/L and <33.45 g/L (Supplementary Figure S2).

### Subgroup analysis for the association between different ALB levels and 28-day mortality

**Figure 5:**
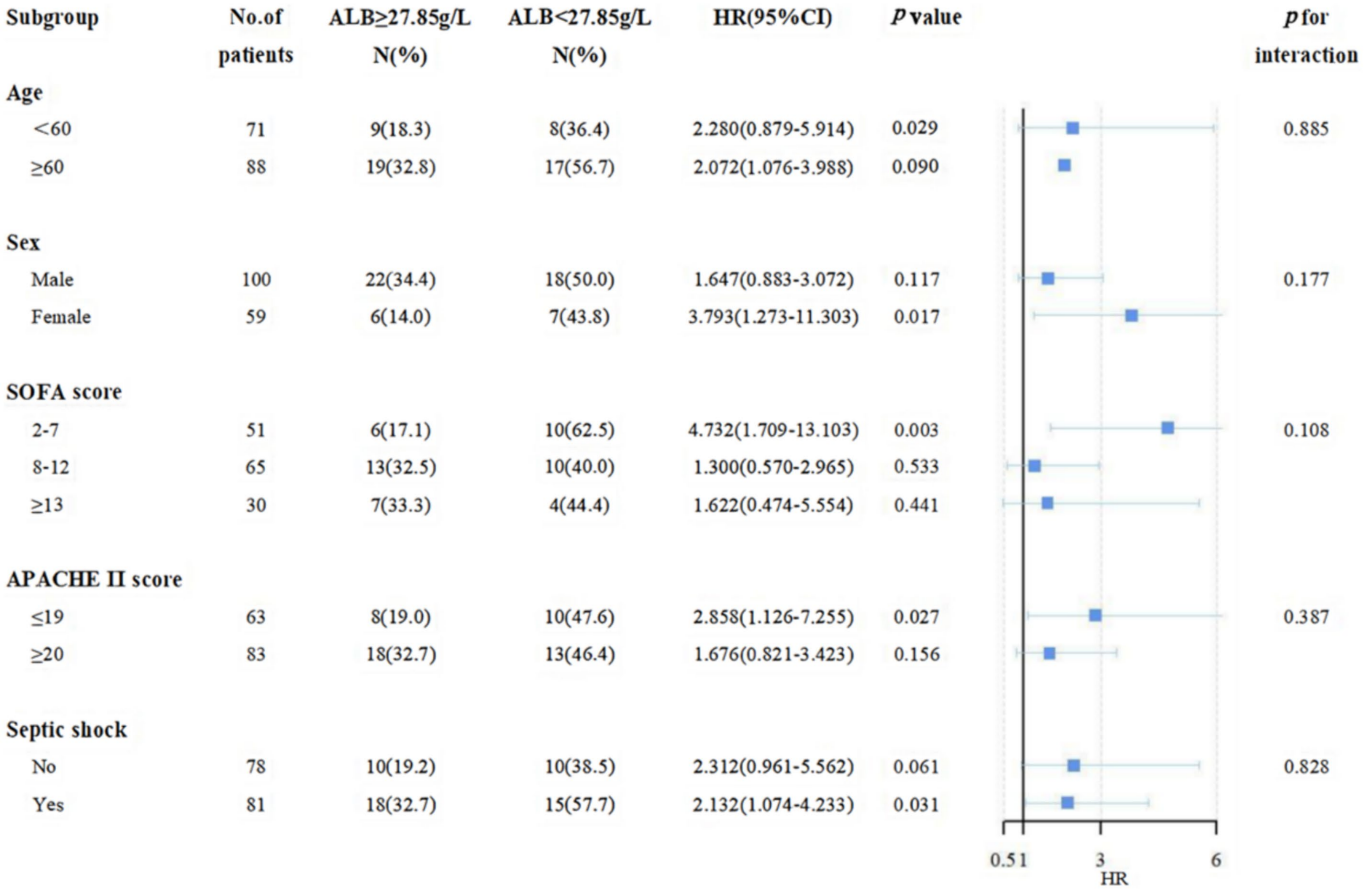
The association between different ALB levels and 28-day mortality in subgroups.

As shown in Figure 5, a negative association between ALB level ≥27.85 g/L on day 7 and 28-day mortality was displayed. In the stratification analysis, among septic patients with ALB level ≥27.85 g/L on day 7, a significant reduction in 28-day mortality and incidence of septic shock was displayed in female patients, under 60 years, and in the low-risk subgroup (SOFA score less than 7, APACHE II score less than 19). Similarly, we also found a negative association between 14-day maximum ALB level ≥33.45 g/L and 28-day mortality in the subgroup analysis (Supplementary Figure S3).

## Discussion

The findings of this study confirmed an association between low ALB levels and mortality, which is consistent with the findings of the majority of the studies we discussed earlier (24, 25). Our study found a significant association between 28-day mortality and ALB level on day 7 and the maximum ALB level within the first 14 days after ICU admission. In addition, each of these variables displayed a positive predictive value for mortality using logistic regression. We dichotomized the variables according to the optimal cutoff values identified by the ROC analysis to ease the clinical application of our findings. The optimal cutoff values for the 14-day maximum ALB level and ALB level on day 7 were 33.45 g/L and 27.85 g/L, respectively. We also found a 1.6 increase in the odds of death in sepsis patients with a 14-day maximum ALB level below the cutoff and a 1.8 increase in the odds of death with the ALB level on day 7 below the cutoff. While our study identifies a potential cutoff for serum ALB levels that may guide ALB infusion, these findings require validation in randomized controlled trials to determine whether targeted ALB supplementation improves outcomes in sepsis patients with hypoalbuminemia.

The 28-day mortality rate of sepsis patients was 36.68% in our study, while global epidemiological sepsis data show that the in-hospital mortality rate of sepsis patients is about 20%. This may be related to the older age of the patients included in our study. Patients older than 65 years accounted for 40.70%. It has been reported that the morbidity and mortality of patients with sepsis increase dramatically with age (2).

Multiple studies have shown that baseline ALB levels are associated with disease prognosis (26, 27), and we also observed lower baseline ALB levels in the non-survival group. While there was no statistical difference in baseline ALB levels between the survival and non-survival groups. We considered that it may be related to the fact that most patients received ALB infusion before admission to our hospital, which narrowed the difference between the two groups. This bias was reduced by developing ALB treatment strategies based on unified decision-making by the clinician after admission. A current study found a significant association between 28-day mortality and 14-day maximum ALB levels, ALB level on day 7, and ALB level on day 14 after ICU admission. Furthermore, the ALB levels were lower in the non-survival group. These results showed an association between ALB levels and disease prognosis, which was consistent with previous research (28, 29).

We found that the optimal cutoffs for 14-day maximum ALB levels and ALB level on day 7 were unique significant independent predictors of 28-day mortality. In addition, each of the predictor variables was a unique, significant predictor of mortality. The probability of living decreased significantly, by 21.91% when the ALB level on day 7 < 27.85 g/L (*p* = 0.007) and by 18.23% when ALB measured within the first 14 days < 33.45 g/L (*p* = 0.008). Thus, it can be seen that ALB may be a useful biomarker in predicting the severity of sepsis. In addition, ALB is a low-cost, readily available measure (30), which may be more convenient for clinical application.

The decrease in ALB levels we observed in septic patients may be related to high levels of oxidative stress and capillary leak (31, 32), which is consistent with the dysregulated host response at the core of sepsis pathophysiology (33). Low ALB exacerbates organ dysfunction by impairing osmotic regulation, antioxidant capacity, and immune modulation, thereby contributing to mortality. In the present investigation, the level of PCT, CRP, WBC counts, NEUT, NLR, and SII showed downward trends, especially in the group with ALB levels above the optimal cutoff value, indicating reduced inflammatory response, which was consistent with previous findings (34). CRP and NLP decreased faster in the group with ALB ≥33.45 g/L, displaying significant differences. It was further confirmed that ALB had antioxidant and anti-inflammatory effects (35, 36).

Sepsis progresses to a severe stage, with refractory persistent hypotension leading to multiple organ dysfunction, that is, septic shock, which has been with a higher fatality rate and a worse prognosis than sepsis (2). In the current study, we found that the incidence of septic shock was lower in the group with ALB above the cut-off value, consistent with previous studies (10, 19, 25). Patients with sepsis are prone to AKI, which increases the mortality rate by 6–8 times (37). In our study, the death group had a higher incidence of AKI. Low levels of ALB are a significant independent predictor of AKI and death following AKI (38). Although no statistical difference, the probability of AKI in the group with higher ALB levels tended to be lower. In addition, respiratory failure is the most common complication of sepsis (4). A randomized controlled study has shown that ALB level works as an independent risk factor for acute respiratory distress syndrome (ARDS) in patients with severe infection. Low ALB levels can prolong mechanical ventilation time and significantly increase mortality (34). In this study, the group with ALB above the optimal cut-off value had longer ventilator-free days, which is similar to the previous studies.

Hypoalbuminemia, or low levels of ALB (often defined as < 3.5–4.0 g/dL or ≤ 3.5 mmol/L), is a well-established risk factor for increased morbidity and mortality (32, 39) and has also been associated with mortality of sepsis; it is modifiable by ALB infusion. Considering the trend of inflammatory indicators and prognosis, we suggest that baseline ALB should not be used as a guide for ALB infusion. The 14-day maximum ALB after admission can be used as a clinical indicator to improve short-term prognosis, and 33.45 g/L is the target threshold to be pursued, which is also close to the threshold for hypoproteinemia. In addition, the earlier the ALB level is raised to a certain level, the higher the 28-day survival rate of patients with sepsis. Hence, the optimal cut-off value for the ALB level on day 7 was also very important, it is recommended to get ALB levels to 27.85 g/L as soon as possible. Moreover, in the stratification analysis, among septic patients with ALB level ≥27.85 g/L on day 7, there was a lower 28-day mortality and incidence of septic shock in female patients, under 60 years, with SOFA score between 2 and 7, APACHE II score less than 19.

This study also analyzed the association between pathogen species and 28-day mortality in patients with sepsis. Previous literature pointed out that sepsis caused by gram-negative bacteria was more severe than that caused by gram-positive bacteria. In our study, we found that the infection rate of gram-positive bacteria was higher in the non-survival group, which may be partially associated with the application of empirical anti-infective treatment at the early stage of disease, tending to choose the antibiotics covering gram-negative bacteria in severe patients. However, with the increase of clinical invasive procedures, the incidence of gram-positive bacteria-induced sepsis is gradually increasing. Studies have shown that the infection rate of gram-positive bacteria in sepsis patients has exceeded that of gram-negative bacteria (2), and the pathogenesis of gram-positive sepsis is still unclear and needs further research.

The close association of PLT count with a prognosis of sepsis has been reported previously (40). PLTs below 50 × 10^9^/L are a robust negative marker of prognosis in patients with sepsis and are thought to be caused by platelet activation and depletion (41). Different markers of PLT function have been recognized as biomarkers of sepsis and have been shown to correlate with severity (42). In this study, univariate logistic regression analysis also suggested that PLTs were associated with the 28-day mortality of septic patients, and thrombocytopenia was a risk factor for the disease.

### Limitations

This study has some inevitable limitations that need to be considered. Firstly, the retrospective nature of this study reduced the strength of the results. However, prospective randomized controlled study in critical patients requires many years to reach the sample size providing statistical power; ethical issues are another consideration when pursuing randomized controlled clinical trials in such populations. Secondly, the patients in this study had a low baseline ALB level, and most of the patients received ALB infusion at the clinician’s discretion, so the relationship between the amount of ALB infusion and disease prognosis could not be analyzed. Nevertheless, with the help of logistic regression and ROC analysis, we explore the target threshold of ALB infusion. ALB level affects the prognosis of critically ill patients and may be related to its immune-modulating function and anti-inflammatory effects. Further prospective controlled studies are needed to clarify the causal relationship between the reduction of inflammatory markers and ALB infusion to guide the prevention and treatment of sepsis better. Additionally, the patients were enrolled in one single medical center with a relatively small sample size, and the generalizability of results may be limited. Nevertheless, the results provide evidence for ALB application in sepsis. The next step would be to conduct a study using a larger sample size at multiple hospitals that provides a nationally representative conclusion.

## Conclusion

Collectively, our findings indicate that ALB levels were inversely associated with 28-day mortality. We found that the cutoffs for 14-day maximum ALB levels and ALB level on day 7 after ICU admission were unique significant independent predictors of 28-day mortality. The probability of living decreased significantly by 21.91% when the ALB level on day 7 < 27.85 g/L and by 18.23% when the ALB level measured < 33.45 g/L at any time. Patients in the group with ALB above the optimal cut-off value (33.45 g/L or 27.85 g/L) had lower mortality, faster decline in inflammatory markers, and decreased incidence of septic shock and AKI, so we suggest that 33.45 g/L as the ALB infusion target within 14 days and 27.85 g/L as the ALB infusion target on day 7 after admission.

## Data Availability

The raw data supporting the conclusions of this article will be made available by the authors, without undue reservation.

## Supplementary material

SUPPLEMENTARY FIGURE S1 Changes of inflammatory indicators in septic patients with ALB level higher or less than 33.45g/L.

SUPPLEMENTARY FIGURE S2 The predictive value of 14-day maximum ALB levels for clinical prognosis.

SUPPLEMENTARY FIGURE S3 The association between different ALB levels and 28-day mortality in subgroups.
