# Supplementary figures and images for: On-treatment serum albumin levels can predict 28-day mortality and guide albumin infusion in sepsis patients
Source: Front Med (Lausanne). 2025 May 14;12:1490838. doi: 10.3389/fmed.2025.1490838 (PMC12116570; doi:10.3389/fmed.2025.1490838)

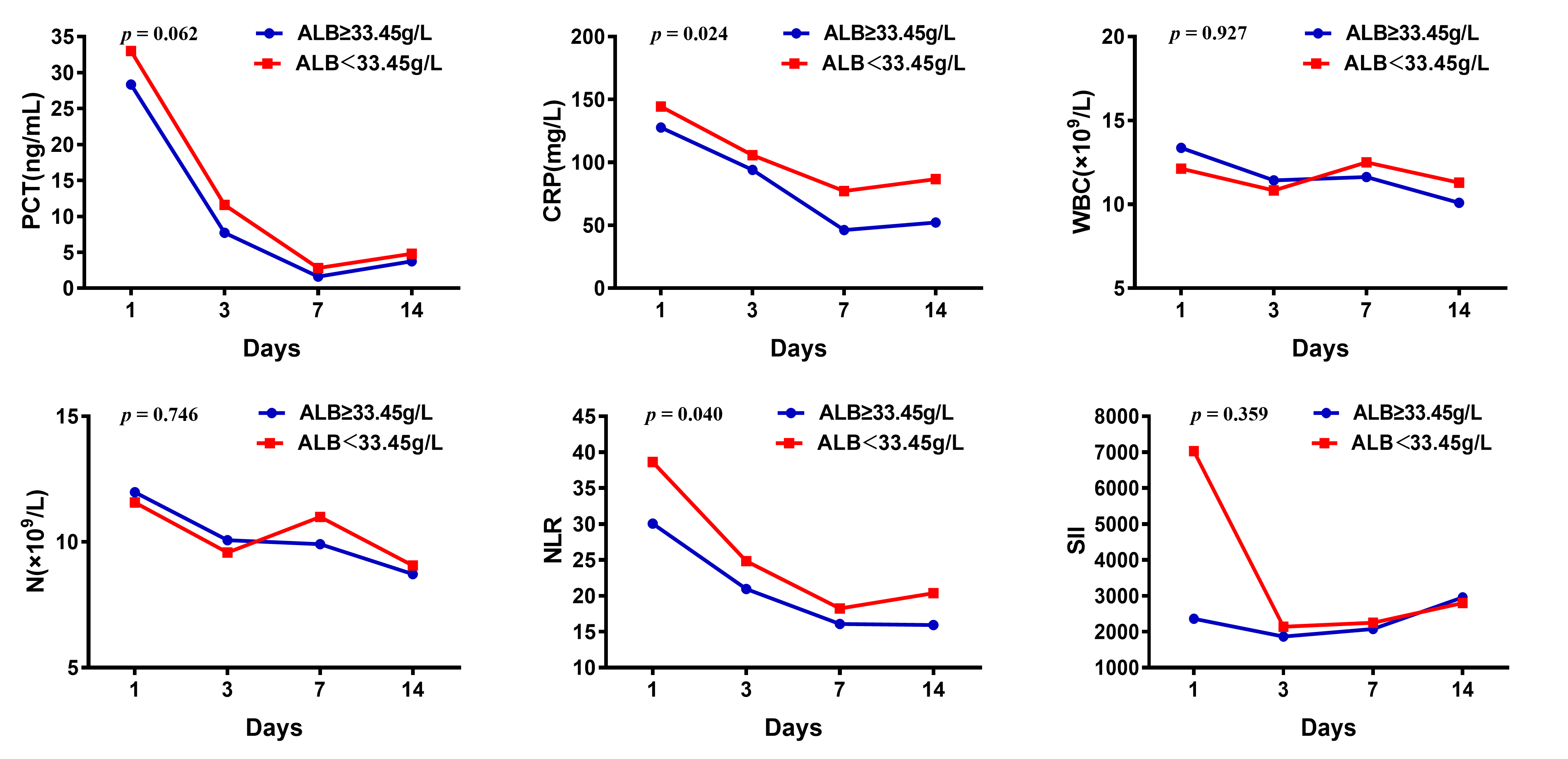

Supplement: SUPPLEMENTARY FIGURE S1 — Changes of inflammatory indicators in septic patients with ALB level higher or less than 33.45g/L. [file Image_1.TIF]

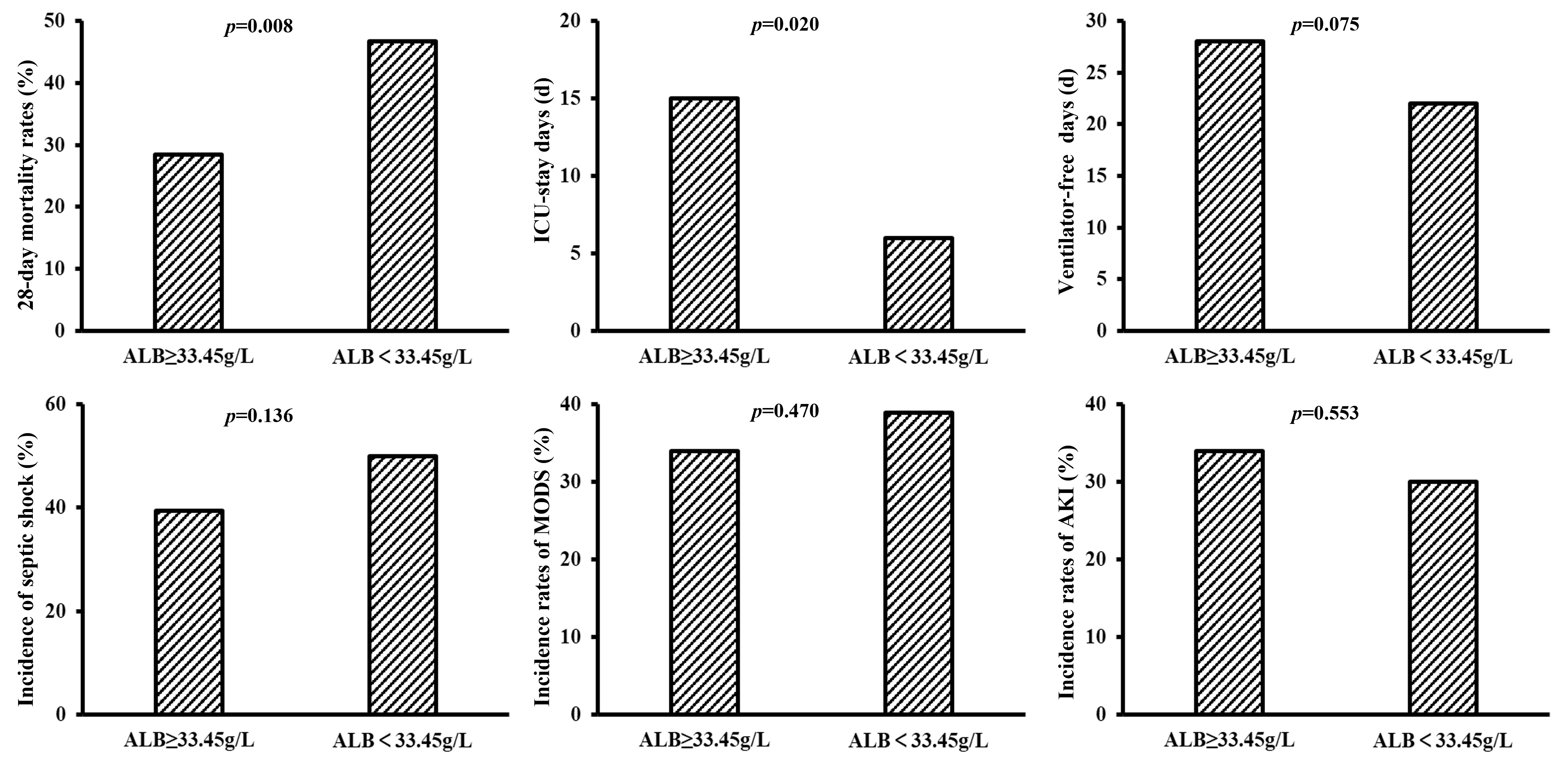

Supplement: SUPPLEMENTARY FIGURE S2 — The predictive value of 14-day maximum ALB levels for clinical prognosis. [file Image_2.TIF]

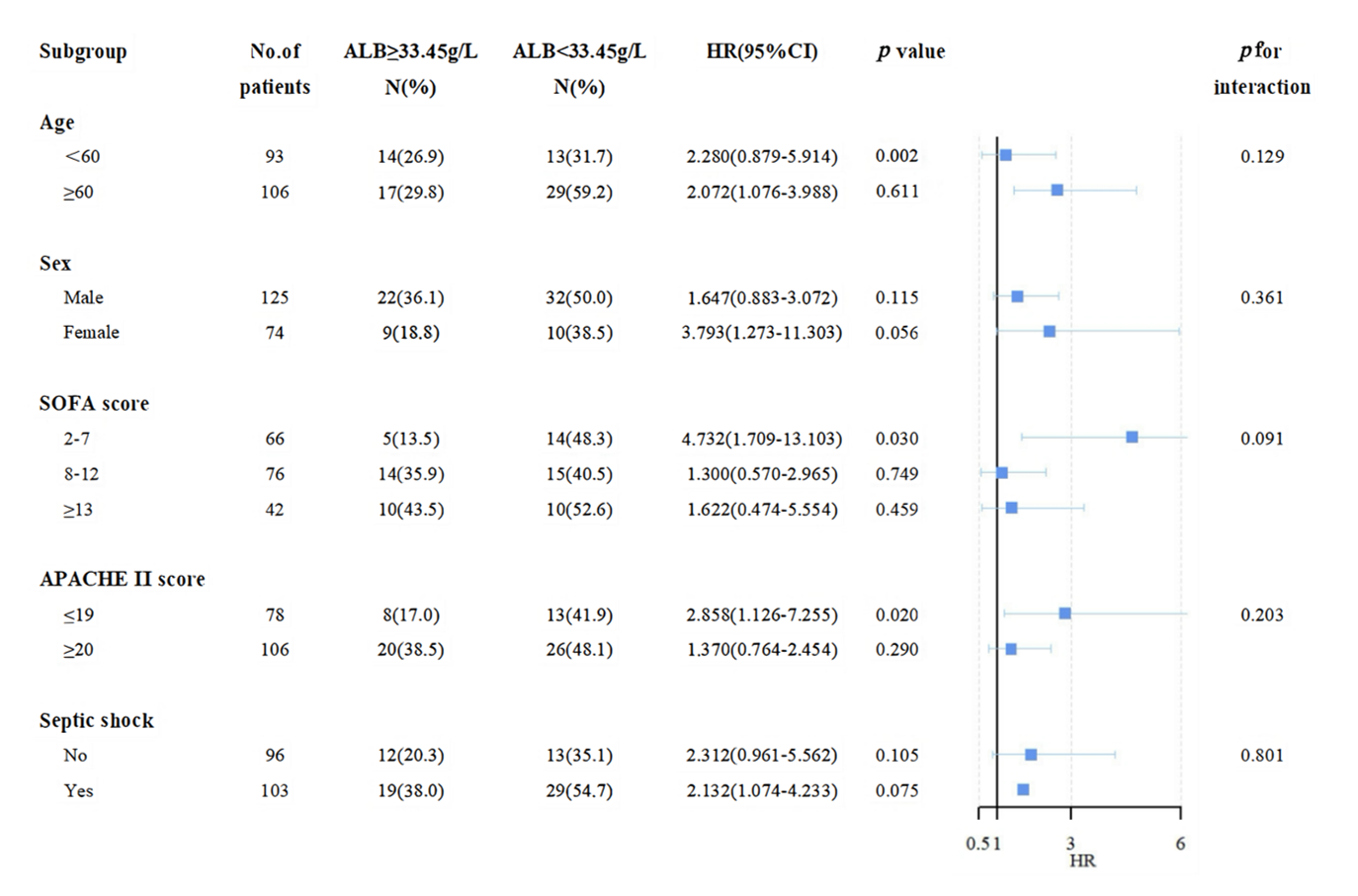

Supplement: SUPPLEMENTARY FIGURE S3 — The association between different ALB levels and 28-day mortality in subgroups. [file Image_3.TIF]
